# Notch Signaling Modulates Macrophage Polarization and Phagocytosis Through Direct Suppression of Signal Regulatory Protein α Expression

**DOI:** 10.3389/fimmu.2018.01744

**Published:** 2018-07-30

**Authors:** Yan Lin, Jun-Long Zhao, Qi-Jun Zheng, Xun Jiang, Jiao Tian, Shi-Qian Liang, Hong-Wei Guo, Hong-Yan Qin, Ying-Min Liang, Hua Han

**Affiliations:** ^1^Department of Pediatrics, Tangdu Hospital, Fourth Military Medical University, Xi’an, China; ^2^Department of Hematology, Tangdu Hospital, Fourth Military Medical University, Xi’an, China; ^3^Department of Genetics and Developmental Biology, Fourth Military Medical University, Xi’an, China; ^4^Department of Biochemistry and Molecular Biology, Fourth Military Medical University, Xi’an, China; ^5^Department of Cardiac Surgery, Xijng Hospital, Fourth Military Medical University, Xi’an, China

**Keywords:** macrophages, notch, signal regulatory protein α, SHP-1, polarization, phagocytosis

## Abstract

The Notch pathway plays critical roles in the development and functional modulation of myeloid cells. Previous studies have demonstrated that Notch activation promotes M1 polarization and phagocytosis of macrophages; however, the downstream molecular mechanisms mediating Notch signal remain elusive. In an attempt to identify Notch downstream targets in bone marrow-derived macrophages (BMDMs) using mass spectrometry, the signal regulatory protein α (SIRPα) appeared to respond to knockout of recombination signal-binding protein Jk (RBP-J), the critical transcription factor of Notch pathway, in macrophages. In this study, we validated that Notch activation could repress SIRPα expression likely *via* the Hes family co-repressors. SIRPα promoted macrophage M2 polarization, which was dependent on the interaction with CD47 and mediated by intracellular signaling through SHP-1. We provided evidence that Notch signal regulated macrophage polarization at least partially through SIRPα. Interestingly, Notch signal regulated macrophage phagocytosis of tumor cells through SIRPα but in a SHP-1-independent way. To access the translational value of our findings, we expressed the extracellular domains of the mouse SIRPα (mSIRPα^ext^) to block the interaction between CD47 and SIRPα. We demonstrated that the soluble mSIRPα^ext^ polypeptides could promote M1 polarization and increase phagocytosis of tumor cells by macrophages. Taken together, our results provided new insights into the molecular mechanisms of notch-mediated macrophage polarization and further validated SIRPα as a target for tumor therapy through modulating macrophage polarization and phagocytosis.

## Introduction

The important roles of microenvironmental elements in tumor development and progression have attracted intensive research attentions recently (1). Macrophages are the predominant population of tumor-infiltrating immune cells and participate in immune regulation in a polarized manner in response to microenvironmental stimuli (2). Classically activated macrophages or M1-polarized macrophages are typically induced by bacterial lipopolysaccharide (LPS) and interferon (IFN)-γ and invoke a type 1 response through secreting cytokines such as interleukin (IL)-12 and increasing antigen presentation capacity (3–5). Moreover, M1-macrophages display a stronger phagocytic activity to “eat” abnormal cells, including dying erythrocytes and mutant cells. On the other hand, M2-polarized macrophages are prototypically activated by IL-4/IL-13 and exhibit phenotypes roughly opposite to M1 macrophages (6). Tumor-associated macrophages (TAMs) share a part of M2 macrophage properties and promote tumor angiogenesis and immune editing through multiple mechanisms (7, 8). Therefore, reprogramming TAMs from M2 into the M1 activation pattern might be a potential therapy for cancers, given that the critical molecular mechanisms controlling M1/M2 polarization are unveiled.

The Notch-RBP-J (recombination signal-binding protein Jk) signaling pathway plays significant roles in macrophage development and activation (9–19). We have recently demonstrated that Notch blockade by RBP-J disruption skews TAMs toward M2 polarization to facilitate tumor progression (10). On the other hand, forced Notch activation by conditional overexpression of Notch intracellular domain (NIC) in macrophages directs TAMs toward an antitumor phenotype by upregulating a few miRNAs (13, 14). Besides, Notch-mediated macrophage polarization also participates in the regulation of phagocytosis and tissue fibrosis (12). However, the downstream molecular mechanisms of Notch signal remain to be elucidated.

Signal regulatory protein (SIRP) α, also known as SIRPA, SHPS-1, p84, and BIT, belongs to the immunoglobulin (Ig) superfamily. SIRPα is abundantly expressed in macrophages, dendritic cells, neutrophils, and neurons (20). Its best characterized ligand is the ubiquitously expressed “don’t-eat-me” signal molecule CD47. When SIRPα binds with CD47 through the Ig-like domains in its N-terminal region, signaling through SHP-1 or SHP-2 is activated in a phosphorylation-dependent manner, followed by a panel of different downstream signaling events (21–23). Kong et al. have demonstrated that SIRPα plays a critical role in regulating innate immune activation (24). Recently, they have further found that SIRPα functions as a significant regulator of TAMs in hepatoma (25).

In this study, we explored the regulatory mechanisms of Notch signal in macrophage polarization. Our results show that Notch signal regulates macrophage polarization at least partly by inhibiting SIRPα. We found that soluble mSIRPα^ext^ polypeptide, a recombinant extracellular fragment of the mouse SIRPα (mSIRPα^ext^), could promote M1 polarization of macrophages and increase their phagocytic activity to L1210 tumor cells in a CD47-dependent way. Our findings extend the molecular signaling mechanisms downstream to Notch in macrophage polarization and highlight SIRPα as a novel target of Notch-mediated macrophage polarization for tumor therapy.

## Materials and Methods

### Animals

Mice were maintained on the C57BL/6 background in a specific pathogen-free facility. RBP-J-floxed (RBP-J^f^) mice (12) or ROSA-Stop^f^-NIC transgenic mice (14) were mated with Lyz2-Cre (#019096, Jackson Laboratory) mice to obtain mice with myeloid-specific Notch blockade (Lyz2-Cre-RBP-J^f/f^, with Lyz2-Cre-RBP-J^+/f^ as a control) or activation (Lyz2-Cre-ROSA-Stop^f^-NIC, with ROSA-Stop^f^-NIC as a control) mice, respectively. Mice were genotyped with tail DNA using polymerase chain reaction (PCR) primers listed in Table S1 in Supplementary Material. All animal experiments were approved by the Animal Experiment Administration Committee of Fourth Military Medical University.

### Cell Culture and Transfection

L1210 murine leukemia cells were cultured in Dulbecco’s Modified Eagle’s Medium (DMEM) (Invitrogen, Carlsbad, CA, USA) supplemented with 10% fetal bovine serum (FBS), 2 mM l-glutamine, 100 µg/mL streptomycin, and 100 U/mL penicillin. Cells were maintained in 5% CO_2_ to 95% air. To culture bone marrow-derived macrophages (BMDMs), mononuclear cells were isolated from tibias and femurs of C57BL/6 mice. Cells were cultured at a density of 2 × 10^6^/mL in DMEM containing 10% FBS and 25 ng/mL murine macrophage-colony stimulating factor (M-CSF) (PeproTech, Rocky Hill, NJ, USA) for 7 days. In some experiments, IFNγ (20 ng/mL, PeproTech), LPS (50 ng/mL, Sigma, St. Louis, MO, USA), or IL4 (20 ng/mL, PeproTech) was added and cultured for 24 h before further analyses. Macrophages treated with PBS, LPS + IFNγ, or IL4 were named as M^PBS^, M^LPS^, or M^IL4^, according to Epelmann et al. (5). Cells were transfected siRNA with Lipofectamine LTX (Invitrogen) according to the recommended protocol. Three pairs of siRNA for each target were designed and their knockdown efficiency was determined by qRT-PCR and Western blotting. The siRNA with highest efficiency was chosen for further experiments. Lentivirus was packaged by Cyagen Biosciences with commercial service (Guangzhou, China).

### Plasmid Construction and Recombinant Protein Purification

Genes encoding the extracellular domain of mouse SIRPα (mSIRPα^ext^) or CD47 (mCD47^ext^) was amplified with PCR using a mouse embryo cDNA library as a template. The primers used were listed in Table S1 in Supplementary Material. The amplified fragments were inserted into pET32a(+) plasmid (Novagen, Darmstadt, Germany), and the expressed fusion protein Trx-mSIRPα^ext^ (55.2 kDa) or Trx-mCD47^ext^ (32.5 kDa) was purified as described previously in Ref. (26). pEFBOS-NIC was as described in Ref. (14).

### Reverse Transcription (RT)-PCR

Total RNA was extracted from cell samples with the Trizol reagent (Invitrogen). cDNA was prepared with a reverse transcription kit (Takara, Dalian, China) following the supplier’s instruction. Quantitative real-time PCR was performed using a kit (SYBR Premix EX Taq, Takara) and the ABI Prism 7500 Real-Time PCR System in triplicates, with β-actin as an internal control. Primers are listed in Table S1 in Supplementary Material.

### Western Blot

Cells were harvested and the whole cell lysates were extracted on ice with the RIPA buffer containing a protease inhibitor cocktail (Beyotime, Haimen, China). Lysates were centrifuged and the supernatants were collected. Protein concentration was determined using a BCA protein assay kit (Pierce). Aliquots of protein lysates were separated by SDS-PAGE and blotted onto polyvinylidene fluoride membrane. Membranes were blocked with bovine serum albumin solution and probed with different primary antibodies and washed, followed by HRP-conjugated secondary antibodies (Table S2 in Supplementary Material). Protein bands were visualized with chemoluminescent reagents (Pierce).

### *In Vitro* Pull-Down Assay

The recombinant Trx-mSIRPα^ext^ protein was cleaved using thrombin (Novagen) in a buffer containing 20 mM Tris–HCl, 150 mM NaCl, and 2.5 mM CaCl2 (pH8.4) at room temperature away from light for 20 h. The ProBond™ purification system (Invitrogen) was used to purify the recombinant mSIRPα^ext^ proteins according to manufacturer’s protocols. The S-tagged mSIRPα^ext^ was mixed with purified Trx-mCD47^ext^ protein (ratio 1:1), and the mixture was incubated at 4°C for 2 h. Then anti-His antibodies (Sigma) pre-coupled to the Dynabeads-protein G (Invitrogen) were added and incubated at room temperature for 30 min with rotation. After washing with PBS-0.02% Tween 20, 50 mM glycine eluent was added and incubated at room temperature for 5 min with rotation. Proteins were collected and analyzed using SDS-PAGE with 15% acrylmide, followed by Western blotting with the anti-S tag and anti-His antibodies.

### Proteomic Analysis

Four-plex iTRAQ-based quantitative proteomics analysis was carried out using proteins isolated from BMDMs of Lyz2-Cre-RBP-J^f/f^ or control mice. Each sample was labeled using iTRAQ 4-plex kits (AB Sciex Inc., Foster City, CA, USA) according to the manufacturer’s instructions. Samples from the control BMDMs were labeled with 114, 115 tags, and samples from the RBP-J knockout BMDMs were labeled with 116, 117 tags, respectively. After labeling, the peptide samples were mixed for further LC-MS/MS analysis. The protein expression level in each sample was quantified and the fold change between control and the RBP-J knockout BMDMs was determined.

### Luciferase Assay

**Figure 1:**
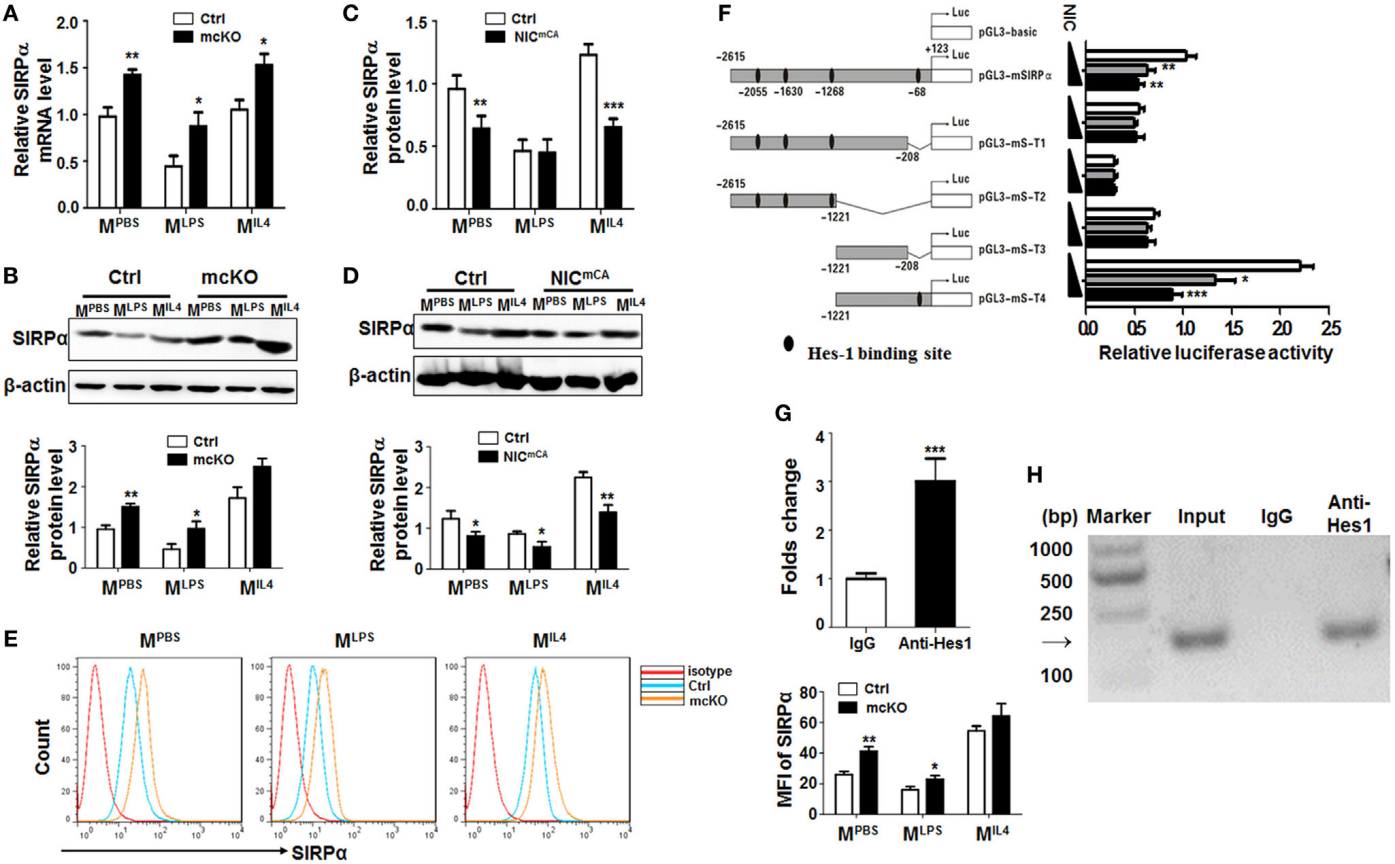
Notch signal regulated Signal regulatory protein α (SIRPα) expression in macrophage polarization. **(A,B)** Bone marrow-derived macrophages (BMDMs) from RBP-J knock out (mcKO) and control (Ctrl) mice were stimulated with PBS, LPS + IFNγ, or IL4 for 24 h. The expression of SIRPα was determined by qRT-polymerase chain reaction (PCR) **(A)** and Western blotting **(B)** (*n* = 4). **(C,D)** BMDMs from Notch signal activation (NIC^mCA^) and control (Ctrl) mice were treated as in **(A)**. The expression of SIRPα was determined by qRT-PCR **(C)** and Western blotting **(D)** (*n* = 4). **(E)** The surface protein level of SIRPα on BMDMs from RBP-J knock out (mcKO) and control (Ctrl) was detected using FACS (*n* = 4). **(F)** Full length and truncated fragments of SIRPα promoter were inserted into pGL3-basic to generate different reporters as depicted. Filled ellipses represent Hes-binding sites. HeLa cells were transiently transfected with different reporters and NIC-overexpressing vector (*n* = 5). **(G,H)** BMDMs were stimulated and subjected to chromatin immunoprecipitation analysis with IgG or anti-Hes1 antibody. Precipitated chromatin DNA was analyzed with qPCR **(G)** or PCR followed by electrophoresis **(H)** (*n* = 4). Student’s *t* test or one-way ANOVA test was used for statistical analyses. Bars represent means ± SD; *P < 0.05; **P < 0.01; ***P < 0.001.

The 5′ flanking sequence (−2,615 to +123) of the murine SIRPα gene was amplified by PCR with mouse genomic DNA as a template. The fragment was inserted into pGL3-basic to generate pGL3-mSIRPα-promoter. Different truncated fragments of the 5′ flanking region, as depicted in Figure 1F, were also generated by PCR and inserted into pGL3-basic (pGL3-mS-T1, 2,3, or 4). HeLa cells (2 × 10^4^) were transfected with different reporters, NIC overexpression plasmid, and phRL-TK using Lipofectamine 2000™ (Invitrogen). The luciferase activity was assessed 24 h later using Luminoskan Ascent (Labsystems, Helsinki, Finland) and a Dual-Luciferase Reporter Assay Kit (Promega) according to the manufacturer’s protocol. All luciferase activity was normalized to the Renilla luciferase activity.

### Chromatin Immunoprecipitation (ChIP)

Chromatin immunoprecipitation assay was performed using a kit (Merck Millipore) according to the manufacturer’s instructions. BMDMs were polarized with LPS + IFNγ for 24 h and fixed with formaldehyde. Cross-linked immune complexes were sonicated and precipitated with anti-Hes1 antibody. DNA was extracted from the collected samples and analyzed by PCR with the primers listed in Table S1 in Supplementary Material.

### Flow Cytometry

L1210 cells (3 × 10^5^) were incubated with mSIRPα^ext^ (10 µg/mL) for 30 min on ice in dark. After washing with PBS, cells were stained with FITC-conjugated anti-S tag antibody (Sigma), followed by FACS analysis using a Calibur™ (BD Immunocytometry Systems, San Jose, CA, USA). BMDMs (3 × 10^5^) were cultured in the presence of PBS (M^PBS^) or LPS + IFNγ (M^LPS^) for 24 h, and then incubated with mSIRPα^ext^ (10 µg/mL) at 37°C for 2 h. After washing, cells were stained with anti-Ki67 (Sigma) or anti-Annexin V (BD), followed by FACS analysis. Data were analyzed using the Flowjo™ software.

### *In Vitro* Phagocytosis Assay

L1210 cells were labeled with carboxyfluorescein succinimidyl amino ester (CFSE, Dojindo Molecular Technologies, Inc.) according to the recommended protocol, and loaded onto macrophages. In some cases, L1210 cells were pre-incubated with purified recombinant proteins at the concentration of 10 µg/mL at 37°C for 2 h before coculturing with macrophages. Cells were stained with anti-F4/80, rinsed with PBS, and visualized under a fluorescence microscope (BX51, Olympus). Phagocytosis was quantified by calculating the average number of ingested L1210 cells in macrophages.

### Statistics

Statistical analysis was performed with the Graph Pad Prism 5 software. Student’s *t-*test or one-way ANOVA test was used for statistical analyses. Data were expressed as means ± SD. *P* < 0.05 was considered statistically significant.

## Results

### SIRPα Was Involved in Notch Signal-Mediated Macrophage Polarization

In an attempt to identify Notch downstream molecules involved in macrophage activation, we employed mice with myeloid-specific Notch blockade (Lyz2-Cre-RBP-J^f/f^ or RBP-J mcKO, with Lyz2-Cre-RBP-J^+/f^ as a control) or activation (Lyz2-Cre-ROSA-Stop^f^-NIC or NIC^mCA^, with ROSA-Stop^f^-NIC as a control). In an initial proteomic analysis of RBP-J deficient and control BMDMs using mass spectrometry, we found that the protein level of SIRPα was upregulated in RBP-J-deficient BMDMs (Figure S1A in Supplementary Material). To validate this finding, RBP-J mcKO, NIC^mCA^, and control BMDMs were stimulated with PBS (M^PBS^), LPS + IFNγ (M^LPS^), or IL4 (M^IL4^) (5), and the expression of polarization markers, Hes1 and SIRPα was examined by qRT-PCR or Western blotting. These treatments induced the expression of various macrophage polarization markers (Figure S1B in Supplementary Material). Meanwhile, Hes1 and Hey1 were upregulated in M^LPS^ and downregulated in M^IL4^ macrophages (Figure S1C in Supplementary Material). SIRPα expression was downregulated in M^LPS^ and upregulated in M^IL4^ BMDMs, as well as in RAW264.7 cells treated in the same way (Figure S1D in Supplementary Material). Moreover, we found that RBP-J deficiency led to upregulated SIRPα expression under different stimuli (Figures 1A,B). On contrary, constitutive Notch activation resulted in a tendency of SIRPα downregulation in macrophages (Figures 1C,D). Flow cytometry confirmed that the expression of SIRPα was upregulated in RBP-J deficiency BMDMs (Figure 1E). These data suggested that Notch signal repressed SIRPα expression in macrophages.

There are four Hes recognition sites (−2,615, −1,630, −1,268, and −68 bp) in the murine SIRPα promoter, suggesting that Notch signal might regulate SIRPα expression through Hes family co-repressors that are downstream to Notch receptors. We, therefore, constructed reporter genes with different truncated fragments of the SIRPα promoter, which were inserted into pGL3-basic (Figure 1F). Reporter assay in the presence of NIC overexpression showed that Notch activation could significantly repress luciferase expression driven by SIRPα promoter fragments, and the Hes-binding site at −68 bp was required for Notch activation-mediated repression of the SIRPα promoter (Figure 1F). Indeed, a ChIP assay indicated that anti-Hes1 antibody could significantly pull-down the SIRPα promoter fragment surrounding the Hes1 binding site at −68 bp in M^LPS^ (Figures 1G,H). These data indicated that Notch signal directly inhibited expression of SIRPα through Hes proteins.

### Notch Signal Negatively Regulated SHP-1 Phosphorylation Triggered by SIRPα–CD47 Interaction

**Figure 2:**
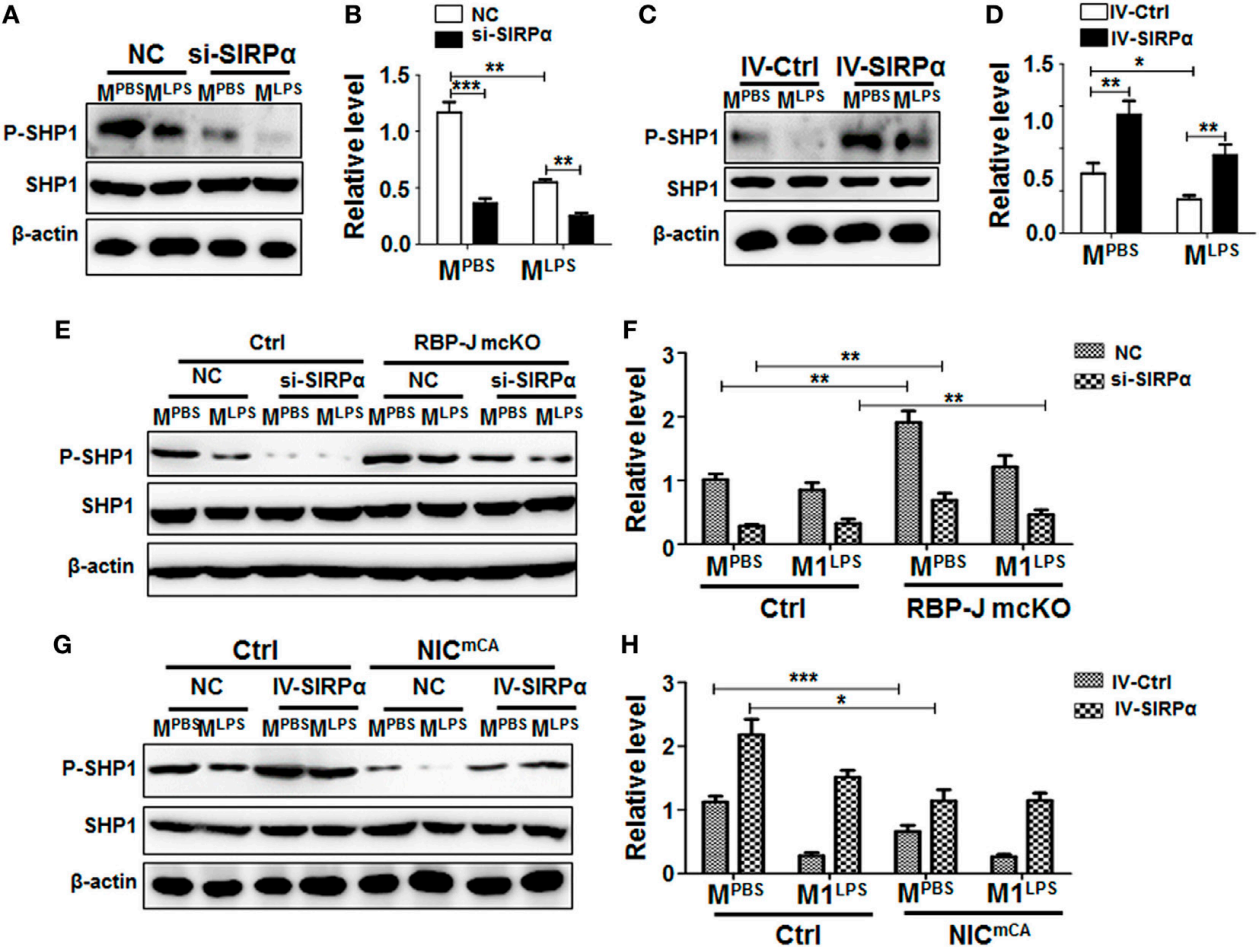
Notch signal regulated signal regulatory protein α (SIRPα) expression and SHP-1 activation. **(A–D)** Bone marrow-derived macrophages (BMDMs) with SIRPα knockdown **(A,B)** or overexpression **(C,D)** were stimulated with PBS or lipopolysaccharide (LPS) + IFNγ for 24 h. Western blotting was carried out to analyze SHP-1 phosphorylation (*n* = 4). **(E,F)** BMDMs from RBP-J deficient (mcKO) and control (Ctrl) mice were transfected with SIRPα siRNA or NC and stimulated with PBS or LPS + IFNγ for 24 h. The phosphorylation of SHP-1 was determined by Western blotting (*n* = 4). **(G,H)** BMDMs from Notch signal activation (NIC^mCA^) and control (Ctrl) mice were infected with SIRPα-overexpressing lentivirus and stimulated with PBS or LPS + IFNγ for 24 h. The phosphorylation of SHP-1 was determined by Western blotting (*n* = 4). One-way ANOVA test was used for statistical analyses. Bars represent means ± SD; * or ^#^, *P* < 0.05; ** or ^##^, *P* < 0.01; ***P < 0.001.

Signal regulatory protein α is enriched in myeloid cells, while its ligand CD47 is universally expressed. Interaction between CD47 and SIRPα induces SHP1/SHP2 activation through tyrosine phosphorylation in the SH2 domains (27, 28), leading to inhibited macrophage phagocytosis and pro-inflammatory response. BMDMs from normal mice were transfected with SIRPα siRNA (si-SIRPα) or control oligonucleotide (NC), which were proven to inhibit SIRPα expression efficiently (Figures S2A,B in Supplementary Material), and stimulated with PBS or LPS + IFNγ for 24 h. Western blotting showed that knock-down of SIRPα by siRNA (si-SIRPα) significantly reduced SHP1 phosphorylation in M^PBS^ and M^LPS^ macrophages (Figures 2A,B). On the other hand, SIRPα overexpression obviously increased SHP1 phosphorylation in macrophages (Figure S2C in Supplementary Material; Figures 2C,D). We then examined the effect of Notch signal on SIRPα signaling using BMDMs isolated from RBP-J mcKO and control mice and stimulated with PBS, LPS + IFNγ, or IL4. The result showed that Notch signal deficiency significantly increased SHP1 phosphorylation both in M^PBS^ and M^LPS^ macrophages (Figures 2E,F). When the expression of SIRPα was knocked down with siRNA, the RBP-J deficiency-induced increase of SHP1 phosphorylation was canceled (Figures 2E,F). On the other hand, forced Notch activation by NIC overexpression reduced SHP-1 phosphorylation, which was reversed by SIRPα overexpression mediated by a lentivirus (Figures 2G,H). These results indicated that Notch signal could repress SHP-1 activation in macrophages, likely through blocking SIRPα expression.

### SIRPα Signaling Modulated Macrophage Polarization

**Figure 3:**
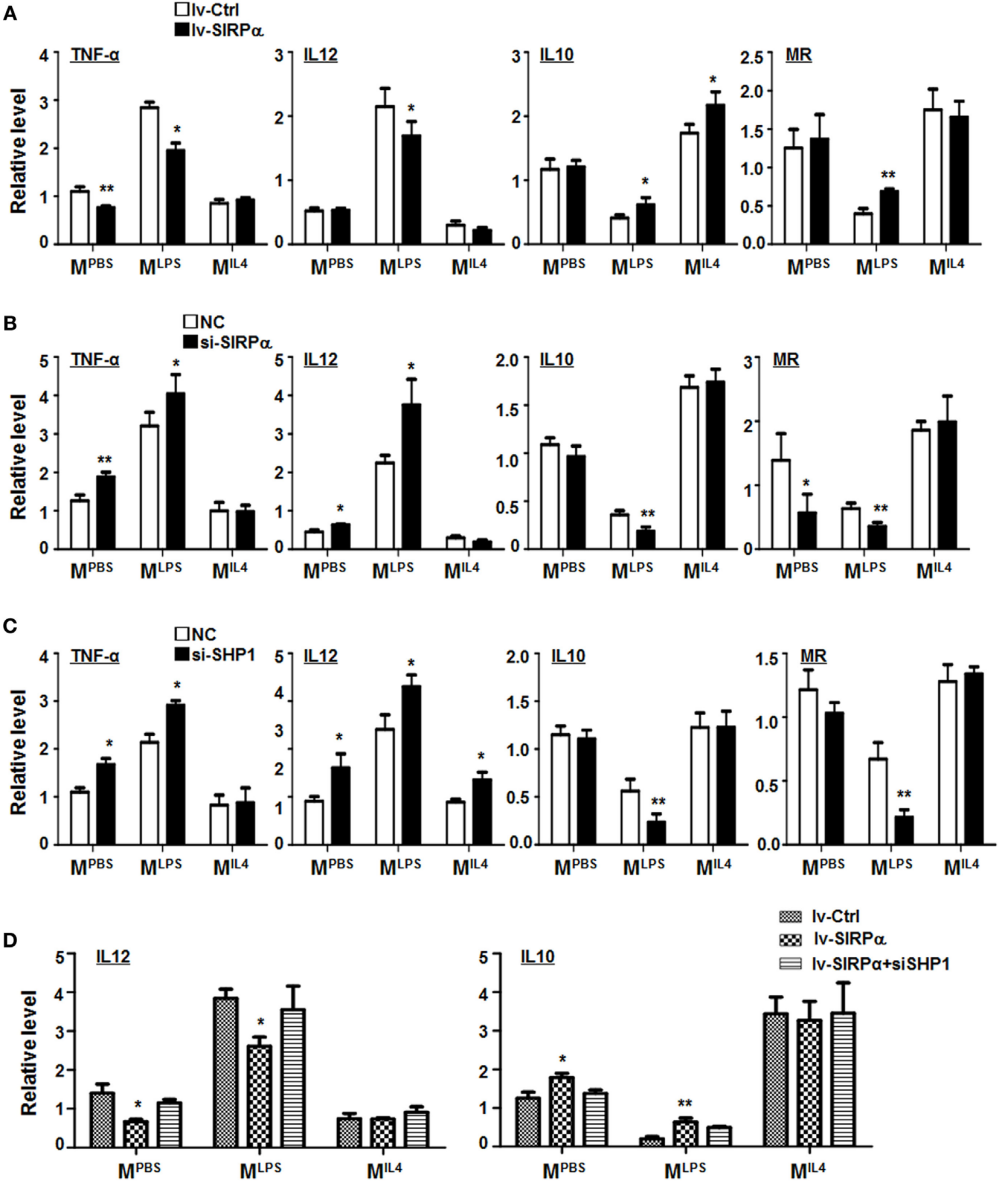
CD47-SIRPα-SHP-1 signal suppressed M1 and promoted M2 polarization. **(A)** Bone marrow-derived macrophages (BMDMs) were transfected with SIRPα or control lentivirus and then cultured with PBS, lipopolysaccharide (LPS) + interferon (IFN)γ, or IL4 for 24 h. The expressions of TNF-α, IL12, IL10, and MR were determined by reverse transcription-polymerase chain reaction (PCR) (*n* = 3). **(B,C)** BMDMs were transfected with siRNA for SIRPα **(B)** or SHP-1 **(C)** followed by stimulation with PBS, LPS + IFNγ, or IL4. The expression of polarization makers was determined by qRT-PCR (*n* = 3). **(D)** BMDMs were transfected with SIRPα or control lentivirus and transfected with SHP-1 siRNA, followed by stimulation with PBS, LPS + IFNγ, or IL4. The expression of IL12 and IL10 was detected by qRT-PCR (*n* = 3). One-way ANOVA test was used for statistical analyses. Bars represent means ± SD; *P < 0.05; **P < 0.01.

Signal regulatory protein α was differentially expressed in macrophages under different polarization stimuli (Figure S1D in Supplementary Material), suggesting that SIRPα-mediated signaling might be involved in macrophage polarization. To validate this, BMDMs from normal mice were transduced with SIRPα-overexpressing lentivirus and simulated with PBS, LPS + IFNγ, or IL4. qRT-PCR was carried out to access the expression of polarization markers in BMDMs. The result showed that SIRPα overexpression downregulated the expression of M^LPS^ markers TNF-α and IL12 and upregulated M2 markers IL10 and MR under LPS + IFNγ stimulation (Figure 3A). Moreover, BMDMs from normal mice were transfected with SIRPα siRNA or NC oligonucleotides and simulated as above. The result showed that the M^LPS^ markers were upregulated while the M^IL4^ markers were downregulated upon SIRPα knockdown (Figure 3B). Knockdown of SHP-1 with siRNA exhibited similar effects on macrophage polarization under various stimuli (Figures S2D,E in Supplementary Material; Figure 3C). Furthermore, SIRPα was overexpressed in normal BMDMs using lentivirus and transfected with SHP1 siRNA simultaneously, followed by polarization stimulation as above. qRT-PCR showed that knockdown of SHP1 abrogated the effect of SIRPα overexpression on macrophage polarization (Figure 3D). These data suggested that the SIRPα-SHP1 signaling repressed M^LPS^ polarization upon LPS + IFNγ stimulation.

### SIRPα Partially Mediated the Effect of Notch Signal on Macrophage Polarization

**Figure 4:**
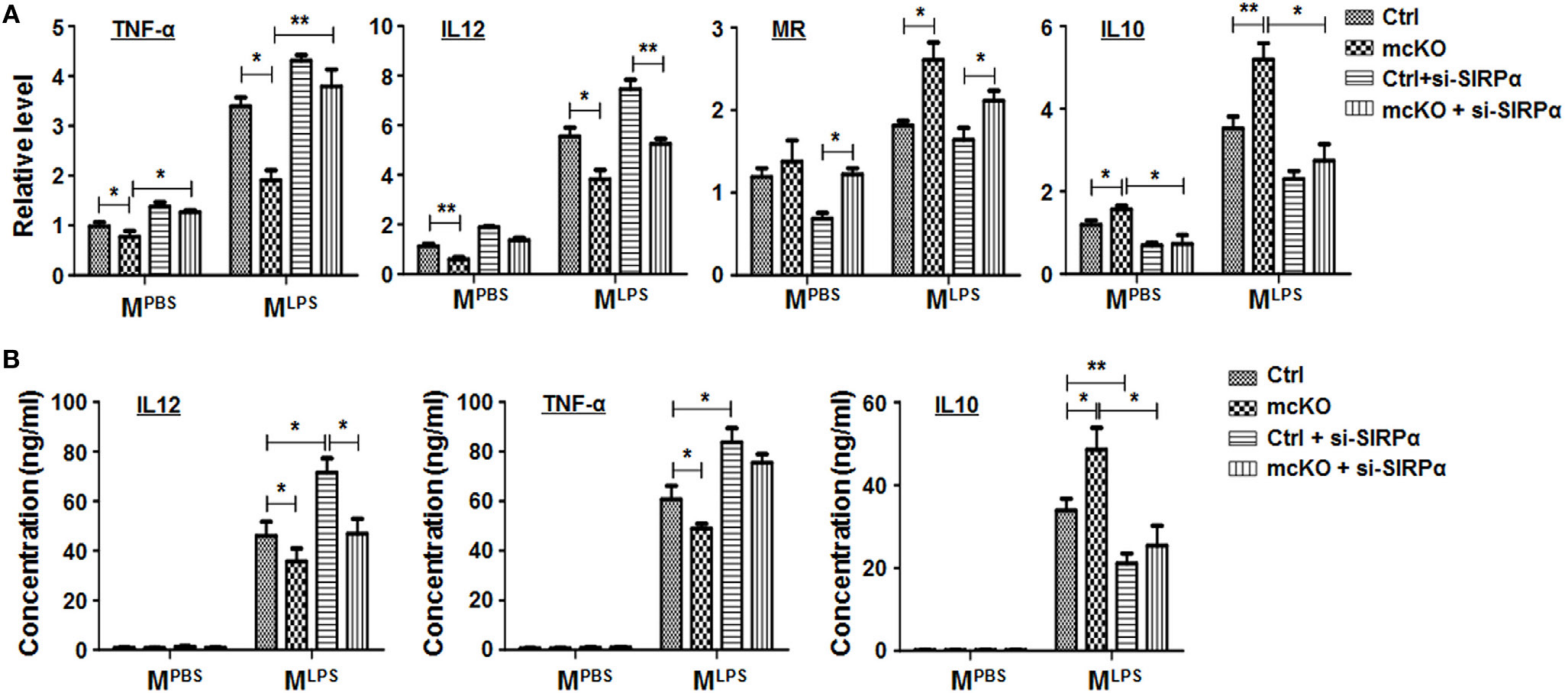
Notch signal regulated macrophage polarization partially through signal regulatory protein α (SIRPα). **(A,B)** Bone marrow-derived macrophagess from RBP-J mcKO and control mice were transfected with siRNA for SIRPα and then cultured in the presence of PBS or lipopolysaccharide + interferon γ for 24 h. The expression of polarization markers **(A)** and the production of cytokines **(B)** were detected (*n* = 4). One-way ANOVA test was used for statistical analyses. Bars represent means ± SD; *P < 0.05; **P < 0.01.

To access the functional significance of SIRPα downstream to Notch signal in regulating macrophage polarization, we cultured BMDMs from RBP-J mcKO mice and treated cells with PBS, LPS + IFNγ, or IL4 in the presence or absence of SIRPα siRNA. qRT-PCR and ELISA were performed to determine the expression of polarization markers and the production of cytokines. The result showed that RBP-J knockout reduced M^LPS^ and increased M^IL4^ marker expression obviously, while knockdown of SIRPα could partially cancel the effects of Notch signal deficiency (Figure 4A). ELISA also showed that blockade of Notch signal suppressed secretion of inflammatory cytokines, such as IL12 and TNF-α, and promoted the production of anti-inflammatory cytokine IL10 (Figure 4B). Similarly, inhibiting SIRPα recovered the cytokine production modulated by RBP-J mcKO (Figure 4B). These data suggested that SIRPα as a downstream molecule of Notch signal could at least partially mediate the effect of Notch signal on macrophage polarization.

### Interaction Between Notch Signal and SIRPα in Regulating Phagocytosis of Macrophages

**Figure 5:**
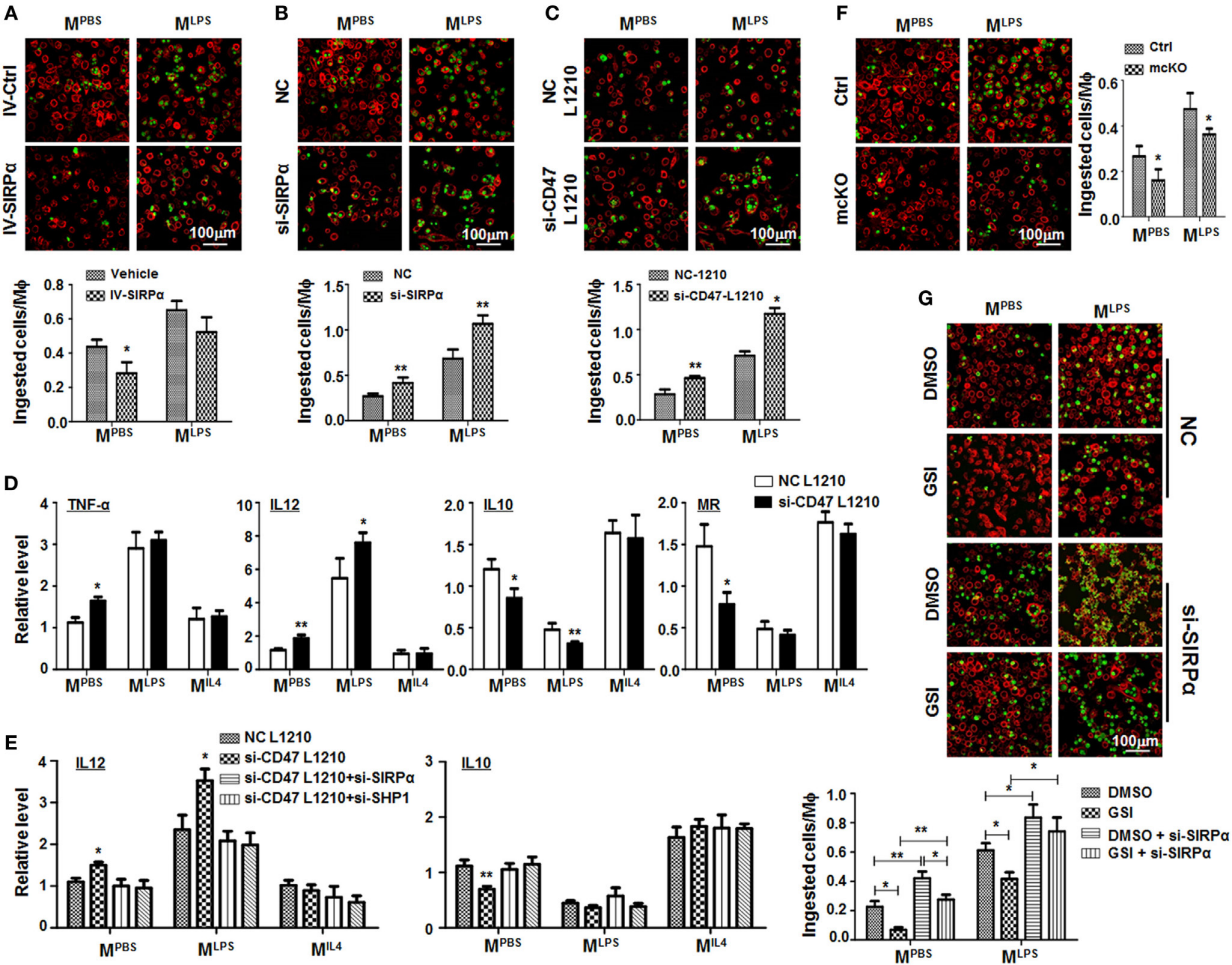
Notch signal increased macrophage phagocytosis through repressing signal regulatory protein α (SIRPα). **(A)** Bone marrow-derived macrophages (BMDMs) were infected with SIRPα overexpression or control lentivirus and stimulated with PBS or lipopolysaccharide (LPS) + interferon (IFN)γ for 24 h. CFSE-labeled L1210 cells were then added and incubated for 2 h. After washing, samples were stained with anti-F4/80 and examined under an immunofluorescence microscope (*n* = 5). **(B)** BMDMs were transfected with si-SIRPα and treated as in **(A)**. The phagocytosis was examined under an immunofluorescence microscope (*n* = 5). **(C,D)** L1210 cells transfected with CD47 siRNA or NC were incubated with PBS- or LPS + IFNγ-stimulated BMDMs. Phagocytosis **(C)** (*n* = 5) and expression of polarization markers **(D)** (*n* = 3) were determined. **(E)** BMDMs were transfected with SIRPα siRNA or NC and stimulated with PBS, LPS + IFNγ, or IL4 and incubated with L1210 cells transfected with CD47 siRNA or NC. The expression of IL12 and IL10 was determined by qRT-polymerase chain reaction (*n* = 3). **(F)** BMDMs from RBP-J mcKO and control mice were treated as in (A). Phagocytosis was examined under an immunofluorescence microscope (*n* = 5). **(G)** BMDMs were treated with DMSO or GSI, and PBS or LPS + IFNγ. Phagocytosis was determined as in **(A)** (*n* = 5). One-way ANOVA test was used for statistical analyses. Bars represent means ± SD; *P < 0.05; **P < 0.01.

To look at the role of SIRPα in macrophage phagocytosis, the L1210 murine leukemia cells were labeled with CFSE, and incubated with PBS-, LPS + IFNγ-, or IL4-stimulated BMDMs for 2 h. The result showed that M^LPS^ BMDMs exhibited increased ability to ingest tumor cells as compared with M^PBS^ or M^IL4^ BMDMs (Figures S3A,B in Supplementary Material). Consistent with previous reports, overexpression of SIRPα in BMDMs using lentivirus attenuated tumor cells engulfment (Figure 5A), and knockdown of SIRPα with siRNA enhanced BMDM phagocytosis (Figure 5B). Consistently, CD47 expressed on L1210 was inhibitory to SIRPα-mediated phagocytosis, because repression of CD47 in L1210 cells significantly increased phagocytosis (Figures S3C,D in Supplementary Material; Figure 5C). Compared with untransfected L1210 cells, incubation with CD47-compromised L1210 cells promoted M^LPS^-like phenotype of BMDMs (Figure 5D), which was dependent on SIRPα or SHP1 expression (Figure 5E). These results suggested that interaction of CD47 on tumor cells with SIRPα on BMDMs regulated both phagocytosis and polarized activation of macrophages.

RBP-J deficiency or treatment with GSI, an inhibitor of Notch signaling, reduced phagocytosis by BMDMs significantly (Figures 5F,G), and this effect of Notch signal blockade was reversed by SIRPα siRNA (Figure 5G). These data suggested that Notch signal increased phagocytosis by macrophages likely through repressing SIRPα.

### Soluble Extracellular Domain of Mouse SIRPα Could Interrupt the CD47–SIRPα Interaction

**Figure 6:**
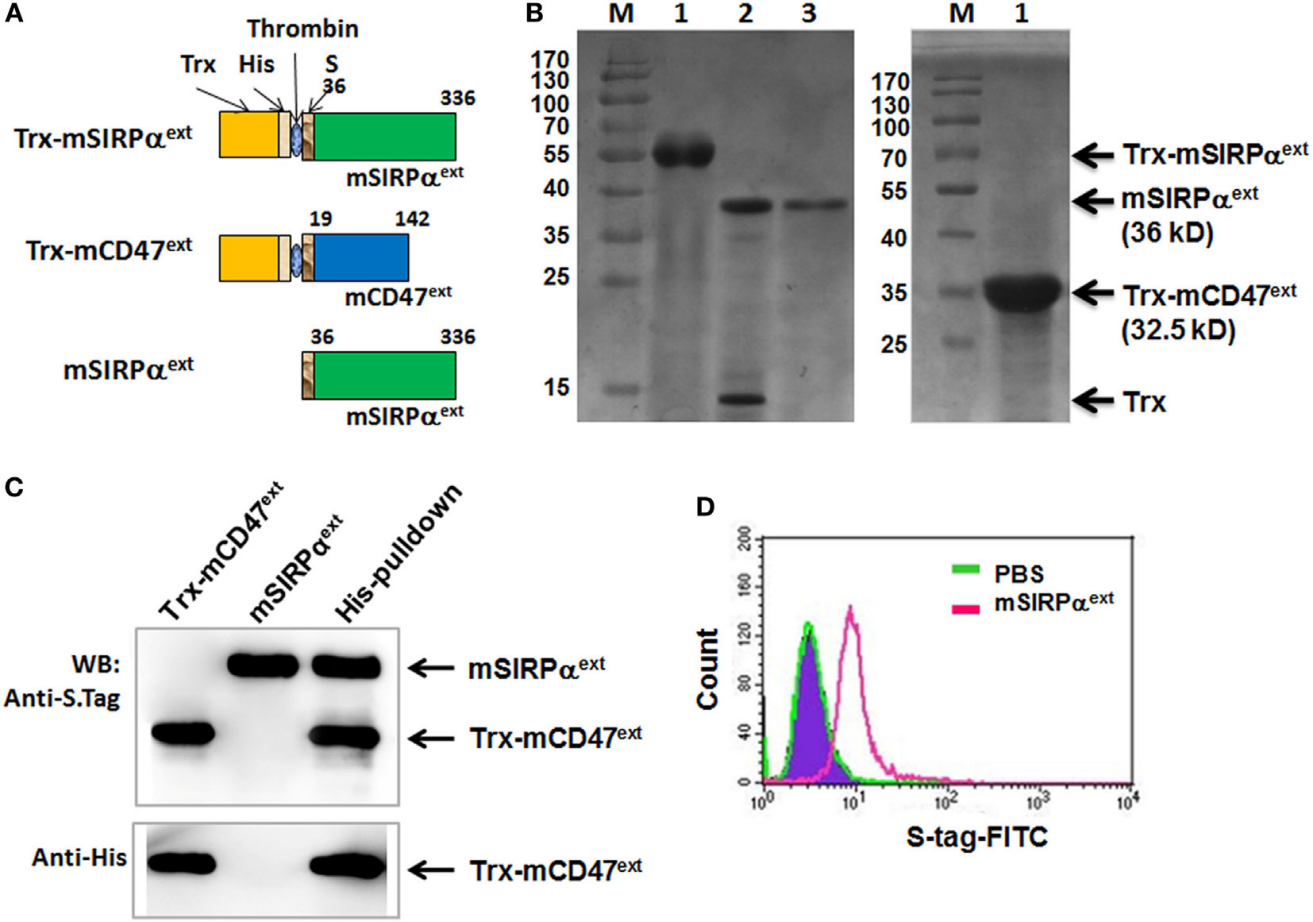
Expression and purification of mSIRPα^ext^ fusion proteins. **(A)** Representative illustrations of the recombinant Trx-mSIRPα^ext^, Trx-mCD47^ext^, and mSIRPα^ext^ proteins. The thioredoxin peptide (Trx), His tag (His), S tag, and the site for thrombin-mediated cleavage are indicated. **(B)** SDS-PAGE of the purified Trx-mSIRPα^ext^ (left, lane 1), cleaved Trx-mSIRPα^ext^ (left, lane 2), purified mSIRPα^ext^ (left, lane 3), and Trx-mCD47^ext^ (right, lane 1). The Trx-mSIRPα^ext^, mSIRPα^ext^, Trx and Trx-mCD47^ext^ bands are indicated with arrows, with MW shown in parentheses. M, molecular weight marker. **(C)** mSIRPα^ext^ interacted with Trx-mCD47^ext^ in a pull-down assay. The purified mSIRPα^ext^ and Trx-mCD47^ext^ proteins were incubated in PBS at 4°C for 2 h and were precipitated with anti-His antibody pre-coupled with Dynabeads-protein G. Co-precipitated proteins were analyzed by Western blotting with anti-His or anti-S Tag antibody. Data represent three independent experiments. **(D)** L1210 cells were incubated with PBS or mSIRPα^ext^. After washing, cells were stained with FITC-conjugated anti-S tag antibody, followed by FACS analysis.

It has been shown that blocking CD47–SIRPα interaction could facilitate phagocytosis of tumor cells and promote antitumor immune response. We have also shown that soluble human CD47 could serve as an antagonist to block CD47–SIRPα interaction (26). Expression vector of the extracellular domain of mSIRPα (mSIRPα^ext^) was constructed and expressed in *E. coli* together with the mCD47^ext^ (Figure 6A). SDS-PAGE analysis of cell lysates revealed that the mSIRPα^ext^ and the Trx-mCD47^ext^ proteins were successfully expressed with predicted molecular weights of 36 and 32.5 kDa, respectively (Figure 6B). Pull-down assay followed by Western blotting showed that the mSIRPα^ext^ protein could be pulled down by the Trx-mCD47^ext^ protein (Figure 6C) but not by Trx (not shown), indicating that mSIRPα^ext^ could bind to mCD47^ext^. To examine whether the mSIRPα^ext^ protein could bind to L1210 cells, we incubated the mSIRPα^ext^ fusion protein with L1210 cells, and analyzed the cells using flow cytometry after staining with a FITC-conjugated anti-S tag antibody. The result showed that cells incubated with mSIRPα^ext^ exhibited significantly higher fluorescence intensity as compared with the control cells (Figure 6D), suggesting that the mSIRPα^ext^ fusion protein could bind to L1210 tumor cells most likely through the interaction with CD47.

### mSIRPα^ext^ Polypeptide Could Promote M1 Polarization and Enhance the Phagocytosis of Macrophages

**Figure 7:**
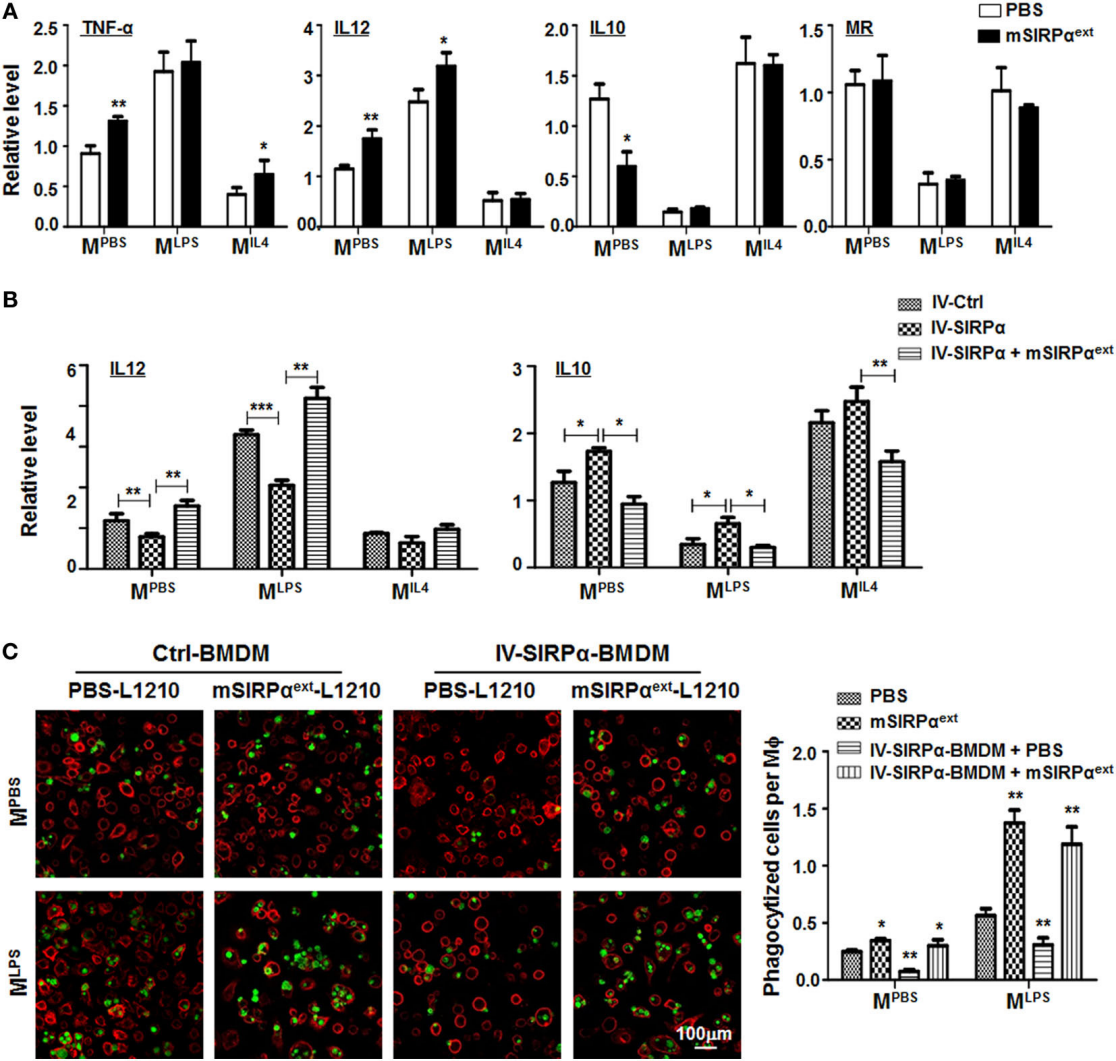
Recombinant mSIRPα^ext^ promoted M1 polarization and enhanced phagocytosis of M1 macrophages *in vitro*. **(A)** Differentially polarized bone marrow-derived macrophages (BMDMs) were incubated with PBS or mSIRPα^ext^ for 6 h. The expression of TNF-α, IL12, IL10, and MR was determined by qRT-polymerase chain reaction (PCR) (*n* = 3). **(B)** BMDMs overexpressing SIRPα were incubated with mSIRPα^ext^ for competitive interaction and stimulated with PBS or lipopolysaccharide (LPS) + IFNγ. The expression of IL12 and IL10 was determined by qRT-PCR (*n* = 3). **(C)** BMDMs were stimulated with PBS or LPS + IFNγ in the presence of mSIRPα^ext^. CFSE-labeled L1210 cells were then loaded and incubated for 2 h, and examined under an immunofluorescence microscope (*n* = 5). One-way ANOVA test was used for statistical analyses. Bars represent means ± SD, *n* = 3; *P < 0.05; **P < 0.01; ***P < 0.001.

To verify whether mSIRPα^ext^ could block the interaction between CD47 and SIRPα and attenuate SIRPα signaling in macrophages, we incubated BMDMs with mSIRPα^ext^ *in vitro*. This treatment did not affect the proliferation or apoptosis of macrophages (Figures S4A,B in Supplementary Material). However, qRT-PCR showed that mSIRPα^ext^ could upregulate the expression of M^LPS^ markers and downregulate that of M^IL4^ marker IL10 (Figure 7A). Moreover, the upregulation of M^LPS^ and downregulation of M^IL4^ markers by mSIRPα^ext^ was canceled by forced overexpression of SIRPα through lentivirus-mediated transfection (Figure 7B). We also examined the phagocytosis of L1210 cells by macrophages in the presence of mSIRPα^ext^. CFSE-labeled L1210 cells were incubated with mSIRPα^ext^ in advanced, and then cocultured with M^PBS^- or M^LPS^-stimulated BMDMs in the presence of mSIRPα^ext^. The result showed that mSIRPα^ext^ could enhance the phagocytosis of macrophages, and overexpression of SIRPα abrogated this effect (Figure 7C). These results suggested that mSIRPα^ext^ fusion protein could block the SIRPα signaling to modulate macrophage activation and phagocytosis, likely *via* competitive interaction with CD47.

## Discussion

The Notch signaling pathway modulates various cell fate determination events during development. Moreover, Notch signal is involved in differentiation and plasticity of hematopoietic cells under both physiological and pathological conditions (29, 30). Indeed, there are four types of Notch receptors in mammals, all of which could induce RBP-J-mediated Notch signal activation. We have shown that Notch1 is the most abundantly expressed Notch receptor in BMDMs (14). Several groups have reported that Notch signal regulates myeloid development and macrophage polarization through multiple downstream molecules, such as SOCS3, cylindromatosis, interferon regulatory factor 8, miR-125a, and miR-148a (9–19). Although these studies have pointed to that Notch signal was required for macrophages M1 polarization, Foldi et al. have demonstrated that Notch signal promotes M2 activation of peritoneal macrophages in an *in vivo* model of chitin-induced M2 polarization (31). This probably suggested that the activation modes of macrophages modulated by Notch signal are context-dependent and influenced by specific pathological processes. Therefore, it should be of significance to further identify other potential downstream molecules of Notch signal. To understand the molecular network modulating macrophage activation downstream to Notch signal, we compared protein expression profiles between wild type and RBP-J deficient BMDMs using iTRAQ and found that SIRPα expression increased in the absence of Notch signaling. qRT-PCR and Western blotting validated the conclusion that SIRPα was downregulated by Notch signal activation or M^LPS^ stimulation in macrophages, while M^IL4^ stimulation or Notch blockade upregulated SIRPα. Further experiments showed that Notch activation repressed SIRPα transcription directly through Hes1-binding sites in its promoter region. Functionally, our data suggested that Notch signal modulated macrophage polarization at least partially through regulating the expression of SIRPα.

Signal regulatory protein α is a myeloid-specific receptor of CD47, which is broadly expressed on many types of somatic cells including tumor cells. SIRPα binds with CD47 and delivers a “don’t eat me” signal for phagocytic cells to help tumor cells escape from immune clearance (32–36). Being activated by signal from adjacent cells *via* cell contact, SIRPα could induce intracellular downstream signaling by a cascade of phosphorylation modifications. The predominant pathway of SIRPα is mediated by SHP-1 activation, which could further influence NF-κB and Akt signaling and then regulate macrophage immune suppression (37, 38). Recent studies have mainly focused on CD47 expression on tumor cells and have developed cancer immunotherapy targeting CD47 (39–41), but the regulation of SIRPα expression and its role in macrophages in tumor microenvironment have been elusive. The present study suggests that SIRPα functions as an important modulator of macrophage polarization. The expression of SIRPα is significantly different in differentially polarized macrophages. Overexpression of SIRPα in BMDMs promoted M2 polarization, while SIRPα knockdown promoted M1 polarization. Interference of CD47 or SHP-1 of SIRPα signaling in BMDMs also promoted M1 polarization, suggesting that SIRPα regulates macrophage polarization dependent on interaction with CD47 and intracellular SHP-1 signaling.

Macrophages have remarkable potential as mediators of anticancer therapies based on their robust ability to ingest tumor cells and modulate tumor microenvironment. The CD47–SIRPα interaction between tumor cells and immune cells represents a critical intercellular communication that inhibits the activation of macrophage-mediated phagocytosis of tumors and thereby acts as a myeloid-specific immune checkpoint (20–23). In this study, we observed that SIRPα overexpression in BMDMs decreased phagocytosis of L1210 leukemia cells, while SIRPα knockdown in BMDMs and CD47 knockdown in L1210 cells both increased phagocytosis by macrophages. Meanwhile, knock down of SHP-1, which modulates M^LPS^ polarization in BMDMs, did not show significant effect on macrophage phagocytosis (data not shown). These findings might suggest that SIRPα-mediated polarization and phagocytosis could be mediated by different mechanisms. Molecular events downstream to these important processes need further studies. In our study, activation of Notch signaling could suppress SIRPα expression and inhibit M^IL4^ macrophage polarization as well as phagocytosis, which might have important implications in tumor microenvironment.

Blocking CD47 triggers the elimination of cancer cells. Nevertheless, the ubiquitous expression of CD47 on normal cells, such as red blood cells, might create a large antigen sink and unintended binding. The unwanted interaction of CD47 on normal cells could be minimized by reducing the binding strength for CD47 (42). On the other hand, high-affinity SIRPα monomers (~14 kDa) are not sufficient to induce macrophage phagocytosis, instead, act as an adjuvant to lower the threshold for phagocytosis in the presence of a separate, tumor-binding antibody. Agents that block SIRPα directly, such as anti-SIRPα antibodies, also act as adjuvants for tumor-binding antibodies (43, 44). To overcome these limitations, we here validated a soluble extracellular domain of mouse SIRPα (36 KD) and confirmed that mSIRPα^ext^ could not only promote M1 polarization but also increase phagocytosis of L1210 leukemia cells by macrophages. Therefore, mSIRPα^ext^ might have a promising potential for the clinical application as a new therapeutic reagent. More studies are needed to assess its future pharmacological and clinical values.

## Ethics Statement

All animal experiments were approved by the Animal Experiment Administration Committee of the Fourth Military Medical University and in accordance with the recommendations of Guide for the Care and Use of Laboratory Animals prepared by the National Academy of Sciences and published by the National Institutes of Health (NIH publication 86-23, revised 1985).

## Author Contributions

HH, Y-ML, and J-LZ designed the research and wrote the manuscript; YL, J-LZ, Q-JZ, XJ, and JT performed experiments; YL, J-LZ, S-QL, H-WG, and H-YQ analyzed data. All authors reviewed and approved the manuscript.

## Conflict of Interest Statement

The authors declare that the research was conducted in the absence of any commercial or financial relationships that could be construed as a potential conflict of interest.
