# Supplementary material for: Notch Signaling Modulates Macrophage Polarization and Phagocytosis Through Direct Suppression of Signal Regulatory Protein α Expression
Source: Front Immunol. 2018 Jul 30;9:1744. doi: 10.3389/fimmu.2018.01744 (PMC6077186; doi:10.3389/fimmu.2018.01744)
Supplement: Supplementary file 1 [file data_sheet_1.docx]

**Supplemental Tables S1-S2**

**Table S1.** Primers and oligonucleotides used for PCR.

| **Name** | **Purpose** | **Sequence** |
| --- | --- | --- |
| Cre-F*  Cre-R*  RBP-J-F  RBP-J-WT-R  RBP-J-floxed-R  NIC-F  NIC-WT-R  NIC-floxed-R  TNF-α-F  TNF-α-R  IL-12-F  IL-12-R  IL-10-F  IL-10-R  MR-F  MR-R  Sirpα-F  Sirpα-R  Actin-F  Actin-R  CD47 siRNA  SIRPα siRNA  SHP-1 siRNA  Hes-1binding-F  Hes-1binding-R  mSIRPα^ext^  mCD47^ext^ | Genotyping  Genotyping  Genotyping  Genotyping  Genotyping  Genotyping  Genotyping  Genotyping  RT-PCR  RT-PCR  RT-PCR  RT-PCR  RT-PCR  RT-PCR  RT-PCR  RT-PCR  RT-PCR  RT-PCR  RT-PCR  RT-PCR  siRNA  siRNA  siRNA  ChIP  ChIP  Cloning  Cloning | 5'-CCGGTCGATGCAACGAGTGATGAGG  5'-GCCTCCAGCTTGCATGATCTCCGG  5'-GTTCTTAACCTGTTGGTCGGAACC  5'-GCTTGAGGCTTGATGTTCTGTATTGC  5'-ACCGGTGGATGTGGAATGTGT  5'-AAAGTCGCTCTGAGTTGTTAT  5'-TAAGCCTGCCCAGAAGACTC  5'-GAAAGACCGCGAAGAGTTTG  5'-CAGGAGGGAGAACAGAAACTCCA  5'-CCTGGTTGGCTGCTTGCTT  5'-GGAAGCACGGCAGCAGAATA  5'-AACTTGAGGGAGAAGTAGGAATGG  5'-CCCTTTGCTATGGTGTCCTT  5'-TGGTTTCTCTTCCCAAGACC  5'-AAACACAGACTGACCCTTCCC  5'-GTTAGTGTACCGCACCCTCC  5'-TCGAGTGATCAAGGGAGCAT  5'-CCTGGACACTAGCATACTCTGAG  5'-CATCCGTAAAGACCTCTATGCCAAC  5'-ATGGAGCCACCGATCCACA  5'-CGAAGTGACAGAGTTATCCAGAGAA  5'-TCGACCGTTCTGAACTGCACTTTGA  5'-TCATCCACCTTAAGTACCCACTGAA  5'-GGCCAGCTAGGAAAGATTT  5'-TTAGACTCCAGTGGGCAAGA  5’-TCGATATCATGGTGACTCAGCCTGAGAAATCAG  5’-AGTGTCGAGTTACTGTTGGTCGTGCTTCACCTGGC  5’-GATATCATGCAACTACTGTTTAGTAACGTCA  5’-TGTCGAGTTAAACCTCCTTTCTCCTCCTCGTA |

**Table S2.** Antibodies and related reagents used in this study.

| **Name** | **Supplier** |
| --- | --- |
| SIRPα  p-SHP-1(Tyr 536)  SHP-1  biotin F4/80  Cy3 avidin  Anti-mouse IgG–FITC  Rabbit polyclonal to S tag  Monoclonal anti-polyhistidine (His)  HRPgoat anti-rabbit IgG  HRP goat anti-mouse IgG | Santa Cruz Biotechnology (for WB)  ebioscience (for FACS)  Santa Cruz Biotechnology  Santa Cruz Biotechnology  eBioscience  eBioscience  Sigma  Sigma  Sigma  Boster Bio Tec  Boster Bio Tec |

**Supplemental Figures S1-S5**

**
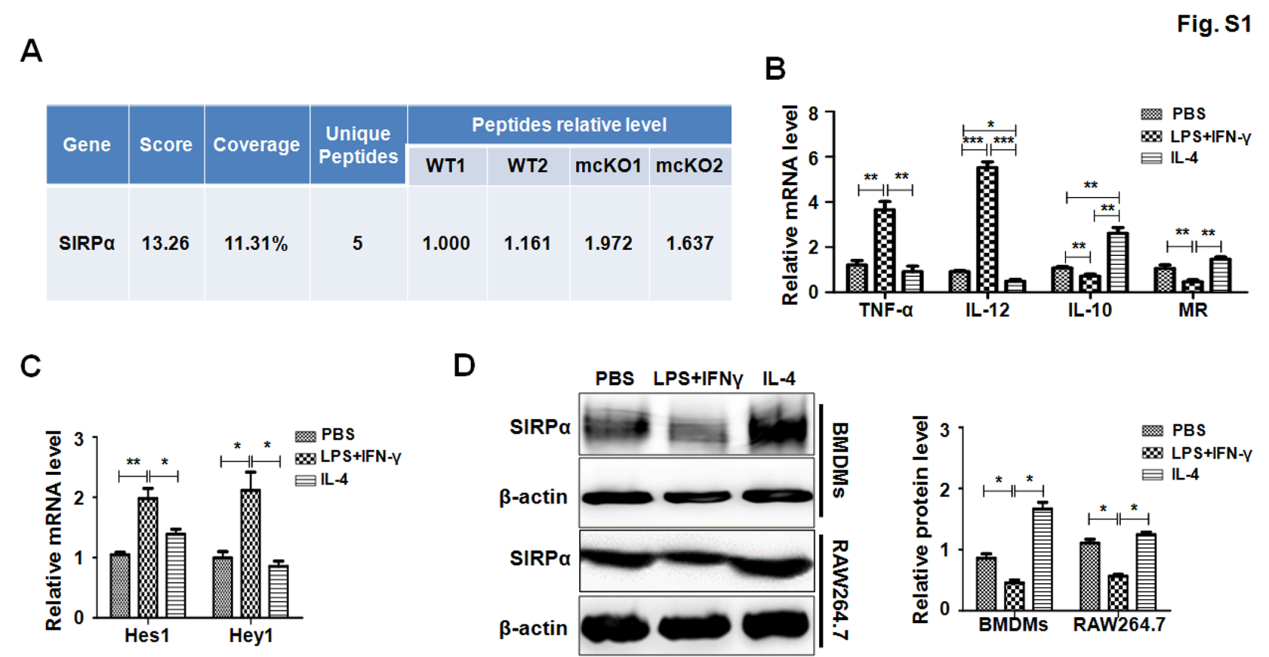
**

**Figure S1. Identification of SIRPα, which was dynamically expressed in macrophages, as a downstream molecule of Notch signaling. (A)** Proteomic analysis was performed with RBP-J mcKO and Ctrl BMDMs using mass spectrometry. The expression of SIRPα was displayed in different groups. **(B, C)** BMDMs were stimulated with PBS, LPS+IFNγ, or IL-4 for 24 h. The expressions of polarization markers (A) and Notch downstream molecules (B) were determined by using qRT-PCR. **(D)** BMDMs or RAW264.7 cells were stimulated with different reagents and the expression of SIRPα was determined by Western blotting. One-way ANOVA test was used for statistical analyses. Bars represent means ± SD, n = 3. *, P < 0.05; **, P < 0.01; ***, P < 0.001.

**
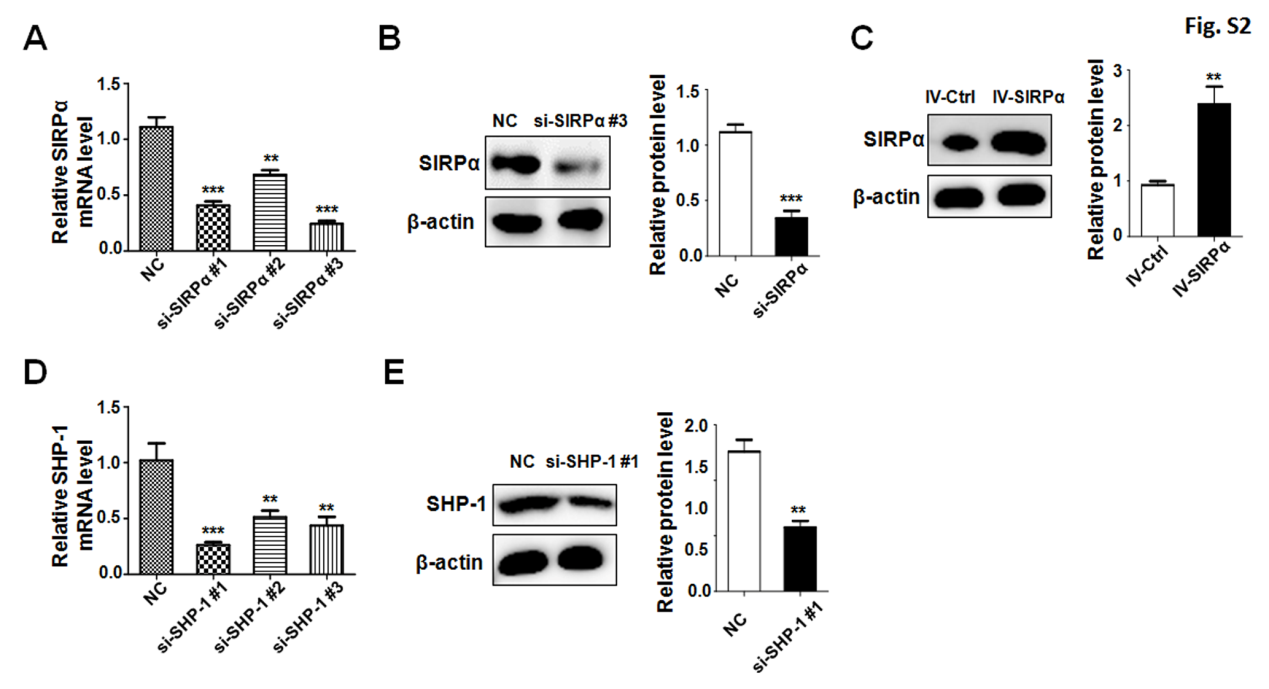
**

**Figure S2. Knockdown or overexpression of SIRPα or SHP-1 in BMDMs. (A, B)** BMDMs were transfected with siRNAs for SIRPα or control (NC). SIRPα mRNA was determined by qRT-PCR (A). SIRPα protein level in cells transfected with si-SIRPα #3 and NC was further detected by Western blotting (B). **(C)** BMDMs were infected with lentivirus to overexpress SIRPα. The SIRPα protein level was detected by Western blotting. **(D, E)** BMDMs were transfected with siRNAs for SHP-1 or control (NC). SHP-1 mRNA was determined by qRT-PCR (D). SHP-1 protein level in cells transfected with si-SIRPα #1 and NC was further detected by Western blotting (E). Student’s *t* test was used for statistical analyses. Bars represent means ± SD, n = 4. **, P < 0.01; ***, P < 0.001.

**
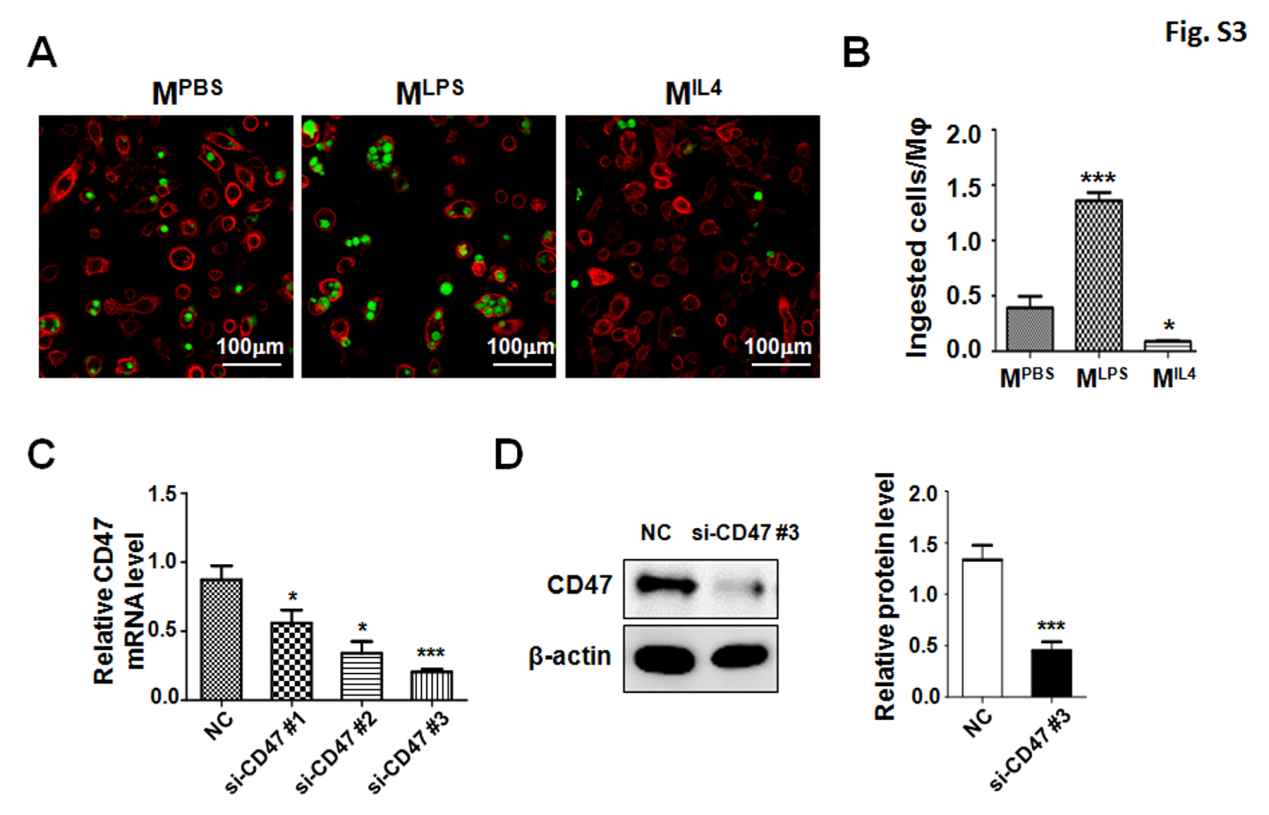
**

**Figure S3. Phagocytosis ability of differentially polarized macrophages. (A, B)** BMDMs were stimulated for different polarization and incubated with CFSE-labeled L1210 cells, and phagocytosis was detected under an immunofluorescence microscope (n = 5). **(C, D)** BMDMs were transfected with siRNAs for CD47 or control (NC). CD47 mRNA was determined by qRT-PCR (C). CD47 protein level in cells transfected with si-CD47 #3 and NC was further detected by Western blotting (D) (n = 4). Student’s *t* test or One-way ANOVA test was used for statistical analyses. Bars represent means ± SD; *, P < 0.05; ***, P < 0.001.

**
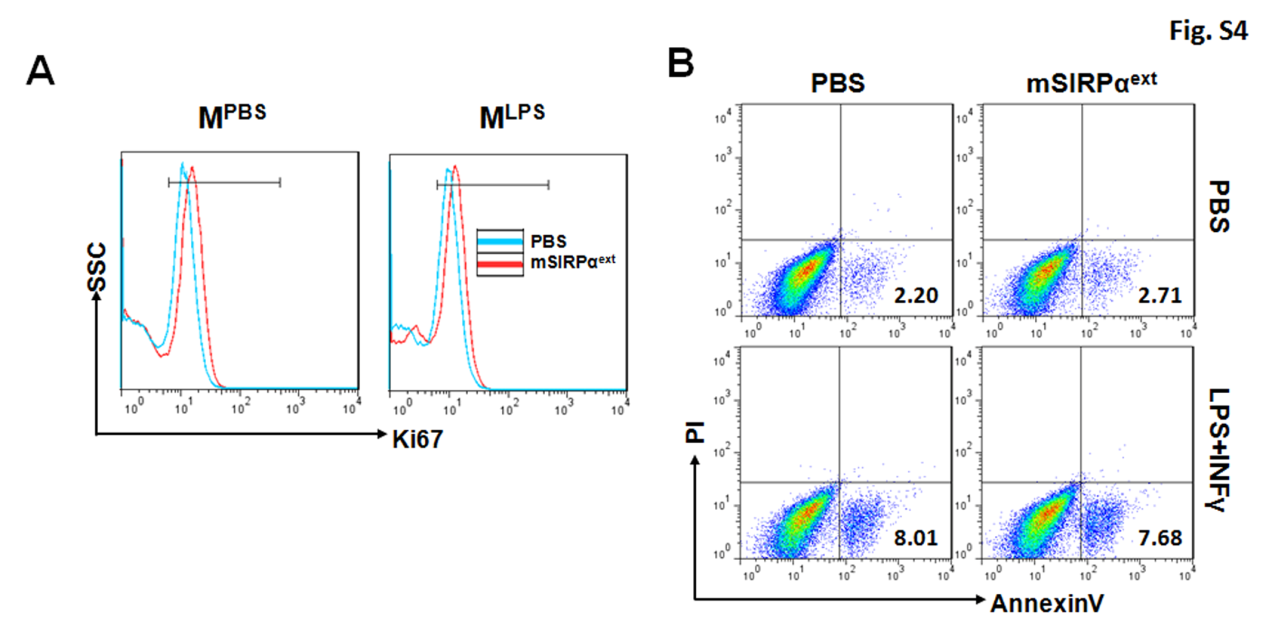
**

**Figure S4. Recombinant mSIRPα^ext^ showed no effect on macrophage proliferation or apoptosis *in vitro*. (A, B)** Differentially polarized BMDMs were incubated with PBS or recombinant mSIRPα^ext^ for 6 h. The proliferation of macrophages was determined using Ki67 staining (A), and apoptosis was determined by using Annexin V-FITC/PI staining (B), followed by FACS analyses.
